# Protracted Hiccups Induced by Aripiprazole and Regressed after Administration of Gabapentin

**DOI:** 10.1155/2021/5567152

**Published:** 2021-04-22

**Authors:** Manuel Glauco Carbone, Claudia Tagliarini, Filippo Della Rocca, Walter Flamini, Giovanni Pagni, Beniamino Tripodi, Donatella Marazziti, Icro Maremmani

**Affiliations:** ^1^Department of Clinical and Experimental Medicine, University of Pisa, Via Roma 57, 56100 Pisa, Italy; ^2^Department of Medicine and Surgery, Division of Psychiatry, University of Insubria, Viale Luigi Borri 57, 21100 Varese, Italy; ^3^Saint Camillus International University of Health and Medical Sciences, UniCamillus-Via di Sant'Alessandro, Roma, Italy; ^4^2nd Psychiatric Unit, Santa Chiara University Hospital, University of Pisa, Italy; ^5^Association for the Application of Neuroscientific Knowledge to Social Aims (AU-CNS), Pietrasanta, Lucca, Italy; ^6^G. De Lisio Institute of Behavioural Sciences, Pisa, Italy

## Abstract

Hiccups are sudden, repeated, and involuntary contractions of the diaphragm muscle (myoclonic contraction). It involves a reflex arc that, once activated, causes a strong contraction of the diaphragm immediately followed by the closure of the glottis translating into the classic “hic” sound. Hiccups can be short, persistent, and intractable depending on the duration. The most disabling hiccups often represent the epiphenomenon of a medical condition such as gastrointestinal and cardiovascular disorders; central nervous system (CNS) abnormalities; ear, nose, and throat (ENT) conditions or pneumological problems; metabolic/endocrine disorders; infections; and psychogenic disorders. Some drugs, such as aripiprazole, a second-generation antipsychotic, can induce the onset of variable hiccups. We describe herein the cases of three hospitalized patients who developed insistent hiccups after taking aripiprazole and who positively responded to low doses of gabapentin. It is probable that aripiprazole, prescribed at a low dosage (<7.5 mg/day), would act as a dopamine agonist by stimulating D_2_ and D_3_ receptors at the “hiccup center” level—located in the brain stem—thus triggering the hiccup. On the other hand, gabapentin led to a complete regression of the hiccup probably by reducing the nerve impulse transmission and modulating the diaphragmatic activity. The present case series suggests the use of low doses of gabapentin as an effective treatment for aripiprazole-induced hiccups. However, our knowledge of the neurotransmitter functioning of the hiccup reflex arc is still limited, and further research is needed to characterize the neurotransmitters involved in hiccups for potential novel therapeutic targets.

## 1. Introduction

Hiccup is the sudden onset of erratic diaphragmatic and intercostal muscular myoclonus with early glottis closure terminating inspiration; hence, the abrupt air rush into lungs induces the vocal cords to close leading to a characteristic “hic” sound [1–3]. Hiccup or “singultus” is a Latin-derived word that means “sob” or “gasp” or rather indicates “the act of catching one's breath while sobbing” [4]. Hiccups are a common experience, representing generally a self-limited disorder that starts with no specific reason and disappears in a few minutes. Many episodes would subside spontaneously without any clinical significance and warrant treatment only when they become persistent and bothersome. If protracted, they can affect conversation, concentration, and oral intake, and they can lead to frustration, fatigue, and insomnia. Severe and prolonged hiccup may lead to exhaustion, fatigue, malnutrition, weight loss, dehydration, and even death in the extreme situations [5, 6].

An individual's hiccup rate is usually consistent for each hiccup episode, occurring at a frequency of 4 to 60 hiccups per minute [7, 8]. The classification of hiccups is by their duration: *acute* hiccups or “hiccup bouts” are of less than 48-hour duration, *persistent* lasts over 2 days, and *intractable* lasts over a month [9]. The incidence and prevalence of hiccups is very difficult to establish as it often represents an epiphenomenon of a wide variety of organic and nonorganic pathologies. Several studies examined the causes of hiccups that have been classified into psychogenic, organic, and idiopathic [10, 11]. Psychogenic hiccups have been reported to occur more commonly in women, although the onset of persistent or intractable hiccups shows a male dominance, mostly over 50 years of age or older [12, 13].

Brief episodes of hiccups are common in healthy individuals, and they are believed to be induced by the rapid stomach distension and irritation in terms of overeating, eating too fast, ingesting spicy food, drinking carbonated drinks, aerophagia, sudden change in ingested food temperature (e.g., hot or cold drinks, a cold water shower, using alcohol, and excessive smoking), or sudden excitement [14, 15].

Persistent and intractable hiccups may signify a more serious underlying etiology such as gastrointestinal and cardiovascular disorders; central nervous system (CNS) abnormalities; ear, nose, and throat (ENT) conditions or pneumological problems; metabolic/endocrine disorders; infections; and psychogenic disorders including excitation, hyperventilation, malingering, somatization, and stress [6, 9, 16–21].

Finally, some drugs, such as chemotherapeutic agents and some glucocorticoids, showed a strong association with hiccups. Nearly 42% of patients taking both cisplatin and dexamethasone may develop hiccups [2, 22]. Interestingly, some of the same medications used to treat hiccups have also, at times, been implicated in their cause (benzodiazepines, opioids, and antidopaminergic drugs); this might be due to the complexity of hiccup origin, possibly involving dopamine (DA), serotonin, opioid, calcium channel, and *γ*-aminobutyric acid (GABA) pathways in the brainstem and medulla [23, 24].

Benzodiazepines show a dose-dependent and inverse relationship with hiccups. At low doses, benzodiazepines correlate with the development of hiccups, while at higher doses, they may be useful in their treatment. Other medications associated with hiccups include alpha-methyldopa, donepezil, levodopa, inhaled anesthetics, ethanol, methohexital, morphine, sulfonamides, tramadol, and azithromycin [11, 22, 25–27].

Although formally known as one of the elective hiccup treatments, recent case reports suggest that antipsychotics (As) may trigger hiccups in some cases [28–31]. Therefore, it seems that, so far, no pharmacological algorithm for the treatment of hiccups has been established. Currently, the US Food and Drug Administration has approved only chlorpromazine, a first-generation A (FGA), to treat hiccups [23]. Another FGA, haloperidol, has been shown to be effective in the hiccup management, possibly via dopamine antagonism. Perphenazine and risperidone have also been indicated as compounds favoring hiccup regression [32, 33].

Anticonvulsants, such as valproic acid, phenytoin, carbamazepine, and gabapentin enhancing central and peripheral GABA transmission, seem to be helpful in blocking the hiccup stimulus. Specifically, gabapentin produces a blockade of neural calcium channels and increases the release of GABA, which might modulate diaphragmatic excitability. Unfortunately, however, only a few data are available to confirm its effectiveness. One study, involving 43 patients with advanced cancer, noted a reduction of hiccups in 95.3% of patients by taking high daily doses of gabapentin (900-1200 mg) [34], with no severe drug interactions.

Other drugs proposed in the treatment of protracted hiccups include defoaming and prokinetic agents (domperidone and metoclopramide), peppermint, proton pump inhibitors, baclofen, nifedipine, methylphenidate, midazolam, lidocaine, and sertraline [23, 24, 35–39].

In drug-induced hiccups, aripiprazole, a second-generation A (SGA), is very often incriminated for transient and persistent hiccups amongst both adult and adolescent patients [27, 28, 30, 40, 41]. Aripiprazole is prescribed for the treatment of adult patients with schizophrenia and bipolar disorder (BD) of type I (BDI) (acute treatment of manic and mixed episodes and maintenance treatment of BDI), in major depressive disorder (as an adjunctive therapy to an antidepressant), and in irritability associated with autistic disorder and Tourette's disorder.

Aripiprazole shows a unique mechanism of action as a type 2 and 3 dopamine receptor (D_2_ and D_3_) partial agonist (as opposed to a full DA antagonist like a SGA), type 1A serotonin (5-HT) receptor (5-HT_lA_) partial agonist, and type 2A serotonin (5-HT) receptor (5-HT_2A_) antagonist. As a partial agonist, aripiprazole acts as a functional antagonist in conditions of high DA concentrations and as a functional agonist in conditions of low DA concentration. This partial agonism might reduce the D_2_ hyperactivation in the mesolimbic pathway, thus alleviating positive symptoms of schizophrenia, but providing enough D_2_-receptor stimulation in the mesocortical pathway and the nigrostriatal pathway, thus preventing, respectively, negative symptoms and extrapyramidal side effects [42]. At the same time, aripiprazole is a well-tolerated drug, especially with regard to metabolic side effects [43].

In the literature, however, there are a few data supporting the effectiveness of some drugs in the hiccups induced by As; however, no guidelines are available for the acute treatment of hiccups induced by aripiprazole.

In the present work, we describe the cases of three BD inpatients who developed protracted hiccups in relation to a treatment with aripiprazole and who promptly responded positively to low doses of gabapentin. At the same time, we will propose the possible neurotransmitter processes underlying the triggering and regression of hiccups.

## 2. Case presentation

### 2.1. Case 1

Mr. A was a 22-year-old man with a diagnosis of schizoaffective disorder and neurodevelopmental disorder not otherwise specified (NOS) according to DSM-5 criteria [44], admitted for the first time in our department, after several previous hospitalizations, for the reexacerbation of a hypomanic episode with dysphoric features and psychotic symptoms.

The patient, a healthy carrier of beta-thalassemia, did not suffer from any other current or past medical conditions and had no family history of psychiatric disorders.

At admission, on psychic examination, the patient presented a slight stutter and logorrheic language with hyperphonic tone. Mimicry was slightly overexpressed with verbal and motor stereotypes. He was euphoric with mild motor restlessness and with reduced need for sleep. Slight acceleration of thoughts with poorly structured paranoid and persecutory ideas.

His longitudinal evaluation revealed that he was born preterm (34th week of gestation due to liver dysfunction from preeclampsia) and acquired walking after the 15th month. Since childhood, the patient has showed some language (expressive and receptive), learning (dysorthography, dysgraphia), and behavioral problems (impulse-control), and since adolescence, he has experienced mood swings, obsessive thoughts (fear of dying and sexual issues), compulsive accumulation, stereotyped language, aggressive attitudes (self-injurious tics), and specific phobias (elevators, fear of drowning, swimming, and cataclysmic events) with serious impairment of personal autonomy and school performance.

In those years, the patient regularly performed several personalized multidisciplinary therapeutic programs in different neuropsychiatric institutes with partial clinical benefit.

In the last three years, Mr. A reported a period of euthymia, achieving the high school diploma and occasionally working as a waiter. However, four months before our consultation, the patient showed mood swings with irritability, nervousness, verbal aggression, and paranoid and persecutory ideas towards parents and colleagues, and he underwent two hospitalizations in other clinics with poor clinical benefit. When he was admitted to our inward, he was taking zuclopenthixol (20 drops/day) and diazepam (15 mg/day).

Lithium sulfate was introduced on the first day, zuclopenthixol and benzodiazepines were gradually suspended until the third day, and aripiprazole (5 mg/day) was given on the fifth day. Approximately 8 hours after aripiprazole, the patient began to experience protracted and bothersome hiccups that lasted for the following 8 hours. The doctor in charge was called by the nursing staff and decided to give him gabapentin (100 mg) that led to a complete hiccup resolution in about 60 minutes.

Since hiccups were considered as an adverse event of aripiprazole, this drug was immediately stopped and replaced by risperidone and clozapine. The patient remained in our hospital for the next 18 days, and he experienced no more hiccups.

### 2.2. Case 2

Mr. G was a 20-year-old man, working in the family business, admitted to our department for the onset of a major depressive episode (according to DSM-5 criteria [45]) with suicidal thoughts and progressive impairment of social and academic functioning.

The family history was positive for anxious spectrum disorders in both maternal and paternal lineages. He referred previous cannabis and alcohol misuse. The patient did not suffer from any medical conditions.

On psychic examination, he was not very talkative, and if questioned, he used short and concise sentences reporting experiences of self-devaluation and inadequacy. Mr. G described a sense of apathy, anhedonia, and a not-better-specified “emptiness.” He referred low energy levels, hypersomnia, difficulties in attention and memorization, poor prospects for the future, suicidal thoughts, and anxiety fluctuations with recent cardiorespiratory and neurovegetative critical episodes. The psychomotricity was slightly slowed. He denies recent alcohol and substance use.

Neurological physical examination did not detect any signs of stiffness or tremor with regular peripheral-induced reflexes.

His longitudinal evaluation revealed no complications at birth, pregnancy, and delivery with regular neuropsychomotor development.

The onset of the psychopathological picture seemed to date back to the age of 14 when the patient experienced a drop in mood with anhedonia, loneliness, feelings of self-devaluation and inadequacy, irritability, anguish, thoughts of death, fluctuations of anxiety levels, and self-cutting. For this reason, he decided to begin a psychotherapeutic treatment with clinical benefit. At the age of 16 years, Mr. G started taking alcohol and cannabinoid for recreational-disinhibitory purposes.

When he was 18, the patient reexperienced depressive symptoms with structured anticonservative ideation and progressive impairment of social and scholastic functioning (he quit the school). He underwent psychiatric evaluations, and several psychopharmacological associations were prescribed including antidepressants, antipsychotics, benzodiazepines, and mood stabilizers with partial clinical benefit. Considering the persistence of the symptomatology, he consulted us, and he was hospitalized in our clinic. At the admission, he was taking valproic acid (50 mg/day, blood levels: 37 *μ*g/mL), perphenazine (4 mg/day), and sertraline (50 mg/day).

On the second day, carbolithium 300 mg/day was added, sertraline was increased to 75 mg/day, and perphenazine was stopped. On fourth day, after increasing carbolithium to 600 mg/day, aripiprazole 5 mg/day was introduced, and the patient reported improvement in mood and anxiety. Aripiprazole was increased to 7.5 mg on the sixth day, and on the subsequent day, about 5 hours after having taken the daily dose of aripiprazole, the patient began to experience hiccups and decided to contact the medical staff (about three hours after the onset). In this case, two doses of 100 mg gabapentin were administered to stop hiccups at a distance of about 2 hours due to the ineffectiveness of the first dose. About 35-40 minutes after the second dose, a complete regression of hiccups was obtained. The medical team stopped aripiprazole, and the hiccup never showed up again.

### 2.3. Case 3

Mr. R was a 41-year-old unemployed man with a diagnosis of BDI and a previous polysubstance use disorder (SUD) (DSM-5) [44]. The patient performed his first hospitalization in our clinic due to the onset of a depressive episode with mixed features.

The patient does not suffer from any other medical illness.

At the admission, the patient was partially accessible to intrapsychic experiences. High reactivity, irritability, and nervousness emerged. He expressed apathy with low interest for work and for social relationships. There are nuanced self-reference and paranoid ideas. He presented an inversion of the sleep-wake rhythm. He described anxiety infradian fluctuations which worsened at evening.

The neurological examination, the routine blood tests, and the toxicological urine test were normal and showed no recent substance use.

The family psychiatric history was positive for mood disorders in the paternal lineage.

The parents described him as extremely vulnerable to life events with high interpersonal sensitivity. Since adolescence, he alternated periods of dynamism, extroversion, and productivity with others of work and social withdrawal.

The onset of the psychopathological picture was traced back to the age of 25, when Mr. R experienced depressive mood with a tendency to isolation and paranoid and persecutory ideas. With family aid, he underwent a first psychiatric consultation, and he took antidepressants (n.s.) with significative clinical improvement.

The following years, the patient achieved a degree in agricultural science and, shortly thereafter, received a teaching job. When he was 34, after the end of a romantic relationship, he experienced a depressive episode again with death thoughts, paranoid ideas, and social and work impairment.

He decided to consult a psychiatrist who prescribed various psychopharmacological associations and progressively regained mood stability and global functioning.

About 6 months before the hospitalization, he presented a reexacerbation of depressive symptoms with paranoid and persecutory ideations toward colleagues, and thus, he decided to contact our clinic.

At the admission, he was taking valproic acid (1000 mg/day), olanzapine (20 mg/day), and biperiden hydrochloride (2 mg/day). Olanzapine was gradually stopped and carbolithium (600 mg/day) was introduced. Aripiprazole (5 mg/day) was added on the third day and increased up to 7.5 mg/day the next day.

Approximately 8 hours after the second aripiprazole, the patient reported the onset of a persistent hiccup. He tried different physical countermeasures, such as holding breath and drinking water while pinching the nose, but none were effective to stop it. Therefore, we decided to administer him 100 mg of gabapentin, and considering the lack of response after about two hours later, an additional 100 mg of gabapentin was given. In less than 60 minutes from the second dose, hiccup regression was observed.

The aripiprazole was discontinued, and the hiccup did not recur again.

## 3. Discussion

The comprehension of hiccups is still being clarified. Current theories include an amorphous neural network coordinating various afferent and efferent pathways, interconnected, coordinated, and managed by a so-called “hiccup center.” The “hiccup reflex arc” consists of three components, the afferent limb including the glossopharyngeal nerve (ninth cranial nerve), vagus nerve (tenth cranial nerve), nuclei of the solitary tract, nucleus ambiguus, phrenic nerve, and sympathetic nerves to convey somatic and visceral sensory signals; the “processing core unit” in the midbrain; and the efferent limb traveling in motor fibers of phrenic nerves to the diaphragm and accessory nerves to the intercostal muscles [1, 46–51]. The central process of hiccups remains poorly understood; probably, the central component is located in the periaqueductal grey matter, subthalamic nuclei among the brain stem respiratory center, phrenic nerve nuclei, reticular formation, and hypothalamus [1, 50]. The reflex arc is potentially mediated by central neurotransmitters (GABA, dopamine, glycine, and serotonin) and peripheral neurotransmitters (epinephrine, norepinephrine, acetylcholine, and histamine) [1]. Any physical or chemical irritants and inflammatory or neoplastic conditions involving the “hiccup reflex arc” may cause hiccups. The complexity of this mechanism, with the involvement of several neurotransmitter systems, explains how many substances can be both triggering and inhibiting hiccups, and at the same time, how the response to a specific treatment can have a high interindividual variability, dependent on many conditions that may influence the neurotransmitter balance of the hiccup reflex arc.

Considering that the only treatment approved by the Food and Drug Administration is chlorpromazine, which has a receptor profile characterized mainly by an antidopaminergic activity acting as a functional antagonist of the D_2_ receptor, it is possible to hypothesize that a hyperdopaminergic state at the central level involves an activation of the hiccup center. This would explain both how other antipsychotics (haloperidol, risperidone, perphenazine, and olanzapine) can be effective in the treatment and how drugs having dopamine-agonist activity can trigger the appearance of simple or protracted hiccups. In fact, many Parkinson disease (PD) patients receiving dopamine agonists have frequent hiccups [52–56]. A retrospective study, including 100 healthy controls and 90 patients with PD, who were constantly receiving antiparkinsonian medications, showed that hiccups were more prevalent in PD individuals than in healthy volunteers (20% versus 3%) [55]. Dopamine agonists share a high affinity for D_3_ receptors that may be involved in the hiccup reflex arc [57]. However, it should be highlighted that, in some other cases, hiccups may occur as the nonmotor symptom of PD rather than as the side effect of anti-PD treatment [55]. Even in some patients, PD was diagnosed after occurrence of intractable hiccups. Hiccups could also be induced by a phrenic nerve activation mediated through agonizing 5-HT_1A_ and antagonizing 5-HT_2A_ receptors [58]. Therefore, it is more plausible that in the genesis of hiccups, there can be an imbalance of multiple neurotransmitter systems, peripherally and/or centrally.

Being a dopamine D_2_ and D_3_ partial agonist, a serotonin 5-HT_lA_ partial agonist, and a serotonin 5-HT_2A_ antagonist, aripiprazole could potentially induce hiccups both through the regulation of the serotonergic and dopaminergic systems. At the serotonergic system level, aripiprazole could enhance the phrenic nerve activity through agonizing 5-HT_1A_ and antagonizing 5-HT_2A_ receptor and consequently trigger the hiccup reflex arc.

Aripiprazole at low doses (approximately up to 7.5 mg) acts as a dopaminergic agonist, stimulating D_2_ and D_3_ receptors. Our cases presented protracted hiccups when they were still taking low doses of aripiprazole, specifically when the antidopaminergic activity had not yet started [43].

Thus, in our case, aripiprazole potentially could have triggered the hiccups through the stimulation of the dopaminergic system by acting as a dopamine agonist and through the serotonergic system by activating the 5-HT_1A_ receptor and antagonizing the 5-HT_2A_ receptor.

On the other hand, gabapentin is an alpha-2-delta ligand, structurally similar to GABA, with the ability to block voltage-operated calcium channels, to reduce the release of several neurotransmitters (glutamate and substance P) and to modulate the diaphragmatic activity [59].

Gabapentin, an alpha-2-delta ligand, structurally similar to GABA, has the ability to block voltage-operated calcium channels, to reduce the release of several neurotransmitters (glutamate, substance P, etc.), and to modulate the diaphragmatic activity [25, 60, 61]. Gabapentin, similar to GABA, functions as an inhibitory mediator at the interneuronal level (presynaptic inhibition) in the brain and spinal cord by altering the transmembrane potential. Inhibition of synapses by GABA has been demonstrated in the cerebellar cortex, hippocampus, olfactory bulb, cuneate nucleus, caudate nucleus, substantia nigra, and septal nucleus and between the vestibular and trochlear motor neurons [62]. Furthermore, it has been postulated in a study that GABA-containing cells in the raphe nucleus are the source of the GABAergic inhibition of the hiccup center [63].

## 4. Conclusion

In the present paper, we described the cases of three men hospitalized in our psychiatric department who experienced the onset of protracted and invalidating hiccups following the intake of a low dose of aripiprazole (<7.5 mg). The hiccups completely disappeared following the administration of low doses of gabapentin and the prompt suspension of aripiprazole. Furthermore, in each case, the hiccups did not recur with drug changes.

This case series would suggest gabapentin as an ideal medication for the acute treatment of aripiprazole-induced hiccups. Given the great use of aripiprazole and considering that hiccups are a common side effect in clinical practice, the administration of low doses of gabapentin might represent an additional tool in the psychiatrist's armamentarium. Gabapentin is a promising and safe drug to treat prolonged, frequent, and persistent hiccups having reported high response rates and few side effects. Furthermore, gabapentin might be considered the first choice in the treatment of hiccups in some psychiatric patients. Indeed, it could be useful in those who are already taking antipsychotic therapy, so any treatment with an additional antipsychotic could lead to a worsening of clinical conditions or in subjects who could be negatively affected by treatment with an antipsychotic from a point of psychomotor and mood view.

However, the exact roles of neurotransmitters within the hiccup reflex arc and the reason why there is a high interindividual variability in response to treatment are still not known. Further research is needed to characterize the neurotransmitters involved in hiccups for potential new therapeutic targets.
